# Cryo-EM structures of CENP-A nucleosomes isolated from human cells

**DOI:** 10.1038/s42003-026-10946-x

**Published:** 2026-09-29

**Authors:** Eisho Tanida, Suguru Hatazawa, Takumi Oishi, Yuki Kobayashi, Mitsuo Ogasawara, Marta A. Jaskiewicz, Akihito Harada, Takeru Fujii, Yasuyuki Ohkawa, Yoshimasa Takizawa, Hitoshi Kurumizaka

**Affiliations:** 1 Laboratory of Chromatin Structure and Function, Institute for Quantitative Biosciences, The University of Tokyo, Tokyo, Japan; 2 Department of Biological Sciences, Graduate School of Science, The University of Tokyo, Tokyo, Japan; 3 Department of Multi-Omics, Graduate School of Medical Sciences, Kyushu University, Fukuoka, Japan; 4 Division of Transcriptomics, Medical Institute of Bioregulation, Kyushu University, Fukuoka, Japan; 5 Department of Computational Biology and Medical Sciences, Graduate School of Frontier Sciences, The University of Tokyo, Tokyo, Japan; 6 RIKEN Center for Integrative Medical Sciences, Yokohama, Japan; 7 Division of Chromatin Structure and Function, Medical Institute of Bioregulation, Kyushu University, Fukuoka, Japan

**Keywords:** Cryoelectron microscopy, Histone variants

## Abstract

Centromeres ensure accurate chromosome segregation, and their identity is specified epigenetically by the histone H3 variant CENP-A. Although the structures of reconstituted CENP-A nucleosomes have provided important insights, how this epigenetic mark is structurally organized within native chromatin remains incompletely understood. Here, we combine cryogenic electron microscopy with chromatin immunoprecipitation (ChIP-CryoEM) to determine the structures of human CENP-A nucleosomes isolated directly from cells without crosslinking or stabilizing agents. We resolved two distinct cellular CENP-A nucleosome structures: a homotypic CENP-A nucleosome and a heterotypic CENP-A/H3 nucleosome, which have been proposed to represent centromeric and ectopic nucleosomes, respectively. Both cellular CENP-A nucleosomes assemble as histone octamers and wrap DNA in a left-handed superhelix, supporting an octameric model proposed for native CENP-A chromatin. In addition, the hallmark CENP-A-specific structural features previously characterized in vitro, including the shortened CENP-A N-terminal α-helix and the distinctive RG loop, are preserved in cellular CENP-A nucleosomes. These findings define the structural organization of CENP-A nucleosomes in cellular chromatin.

## Abstract (web summary)

A study employs ChIP-CryoEM to determine structures of CENP-A nucleosomes isolated from human cells. Homotypic CENPA and heterotypic CENP-A/H3 nucleosomes are octameric and retain hallmark CENP-A structural features.

## Introduction

Faithful chromosome segregation during cell division relies on the centromere, a unique chromosomal locus that ensures proper kinetochore assembly and microtubule attachment. Unlike most chromosomal regions, the centromere is not defined by a specific DNA sequence but by the presence of the histone H3 variant CENP-A, which replaces canonical H3 within centromeric nucleosomes^1–6^. The incorporation of CENP-A is both necessary and sufficient for centromere function, and its propagation across cell generations underlies the epigenetic inheritance of the centromere position^2,7–9^. Despite substantial progress in understanding the CENP-A nucleosome structure and centromere identity maintenance, the specific structural features underlying this process remain incompletely defined.

A long-standing controversy has surrounded the structural nature of the CENP-A nucleosome—whether it adopts a canonical octameric configuration similar to histone H3 nucleosomes or instead forms a noncanonical nucleosome such as a hemisome, composed of single subunits of H2A, H2B, CENP-A, and H4^10–15^. Early biochemical and biophysical studies suggested that the CENP-A nucleosome might exhibit noncanonical, tetrameric arrangements, raising the possibility that hemisomes could represent a centromere-specific nucleosome form^10,13,16^. Subsequent reports proposed that CENP-A nucleosomes might exist as tetramers rather than octamers in certain cell-cycle states^17,18^, whereas other studies argued that the native form adopts the canonical octameric organization^19–22^. These conflicting models have fueled a long-standing debate over the true structural nature of the centromeric nucleosome. Moreover, the overproduction of CENP-A, which is frequently observed in aggressive cancer cells, leads to the ectopic spreading of CENP-A along chromosome arms, where heterotypic nucleosomes containing one CENP-A and one H3 molecule are assembled^23,24^. In addition, CENP-A-H4 particles lacking H2A-H2B have been reconstituted in vitro, further highlighting the structural diversity of CENP-A-containing nucleosomal particles^25,26^.

In vitro reconstitution experiments and structural analyses have provided increasing evidence for an octameric organization^27–33^. Despite this progress, whether the CENP-A nucleosome assembled within human cells truly adopts an octameric or hemisomal architecture has remained unresolved, largely due to the lack of direct high-resolution structures from cellular material. In the present study, our cryogenic electron microscopy examination coupled with chromatin immunoprecipitation (ChIP-CryoEM) enabled the direct visualization of human CENP-A nucleosomes prepared from human cells.

## Results

### Isolation of human CENP-A nucleosomes

**Fig. 1:**
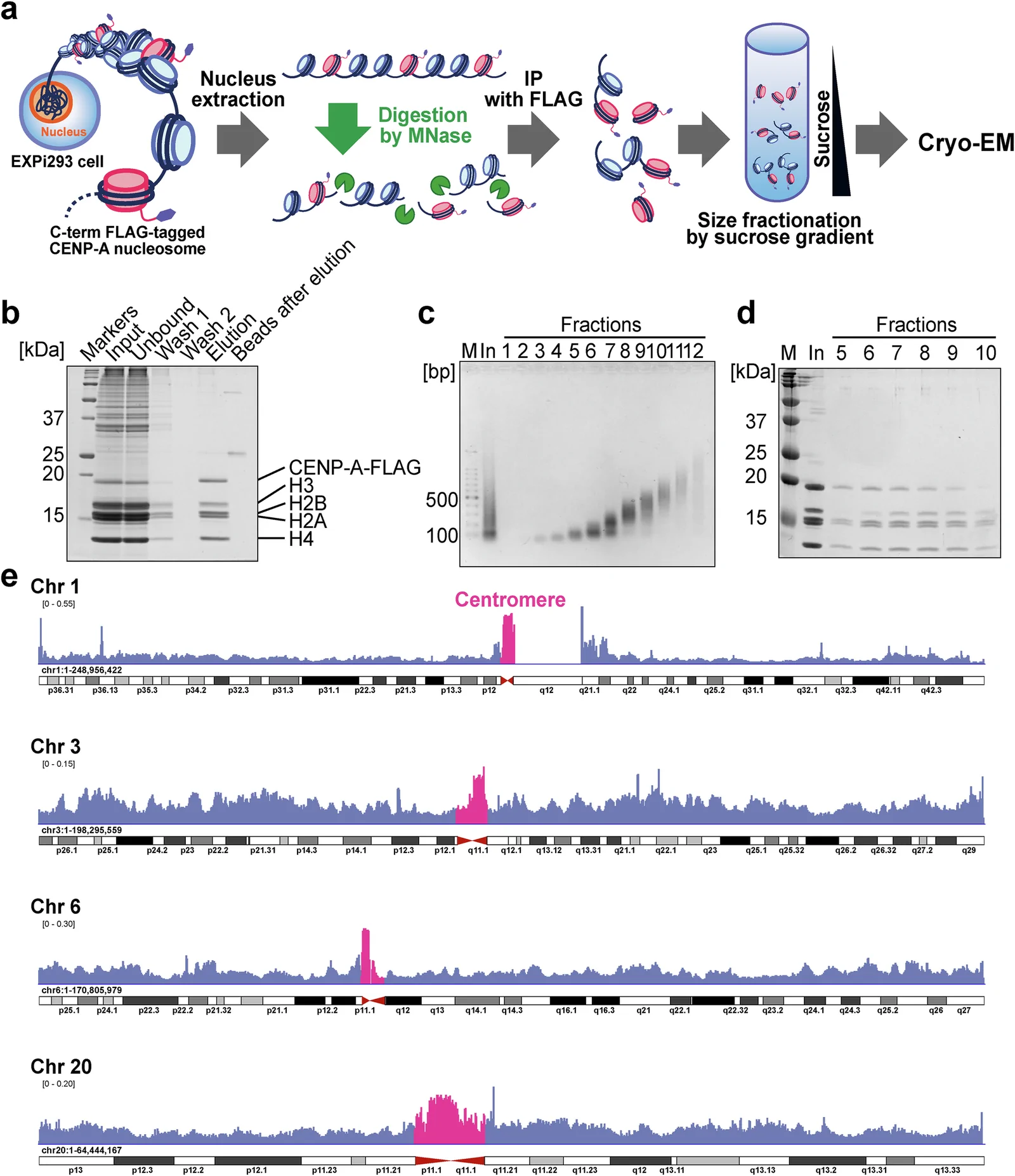
ChIP-CryoEM analysis of CENP-A nucleosome. **a** Schematic overview of the ChIP–cryo-EM analysis of CENP-A nucleosomes. **b** SDS–PAGE analysis of proteins in fractions from the FLAG immunoprecipitation (IP). **c** Native-PAGE analysis of DNA lengths in fractions from the sucrose gradient. **d** SDS–PAGE analysis of proteins in fractions from the sucrose gradient experiment. **e** Representative examples of genome-wide mapping of CENP-A nucleosomes based on sequencing data. Blue signals indicate ectopic CENP-A/H3 peaks along chromosome arms. Although the total amount of ectopic CENP-A/H3 nucleosomes may exceed that of centromeric CENP-A nucleosomes, CENP-A enrichment occurs more frequently at centromeres, and the plot should be interpreted as showing the signal distribution, rather than the total CENP-A abundance.

To investigate the structural organization of human CENP-A nucleosomes in cells, we first isolated CENP-A-containing chromatin using a chromatin immunoprecipitation (ChIP)-based strategy (Fig. 1a). We expressed C-terminally FLAG-tagged CENP-A in Expi293 cells, a derivative of the human embryonic kidney line HEK293. We collected unsynchronized cells, in which the majority were in interphase, and a minor population was mitotic. Nuclei were isolated and subjected to micrococcal nuclease (MNase) digestion under low-salt (100 mM NaCl) conditions, in which MNase preferentially cleaves linker DNA. In this step, the nucleosomes located within MNase-sensitive open chromatin regions were expected to be digested and removed.

The remaining chromatin pellet, which likely contained condensed regions such as centromeric chromatin, was resuspended in high-salt buffer (400 mM NaCl) and digested with MNase a second time. From this high-salt fraction, CENP-A nucleosomes were affinity-purified using anti-FLAG M2-conjugated beads (Fig. 1b), and CENP-A mono-nucleosomes were further fractionated by sucrose-gradient ultracentrifugation (Fig. 1c, d). Genome-wide mapping revealed that the purified CENP-A nucleosomes were enriched at centromere, as well as ectopically spread to chromosome arms (Fig. 1e). These results are consistent with previous studies, in which the overproduced CENP-A was substantially located within the CENP-A nucleosome at the centromeres and at the ectopic loci in chromosome arms^23,34^.

### Cryo-EM structures of CENP-A nucleosomes isolated from human cells

**Fig. 2:**
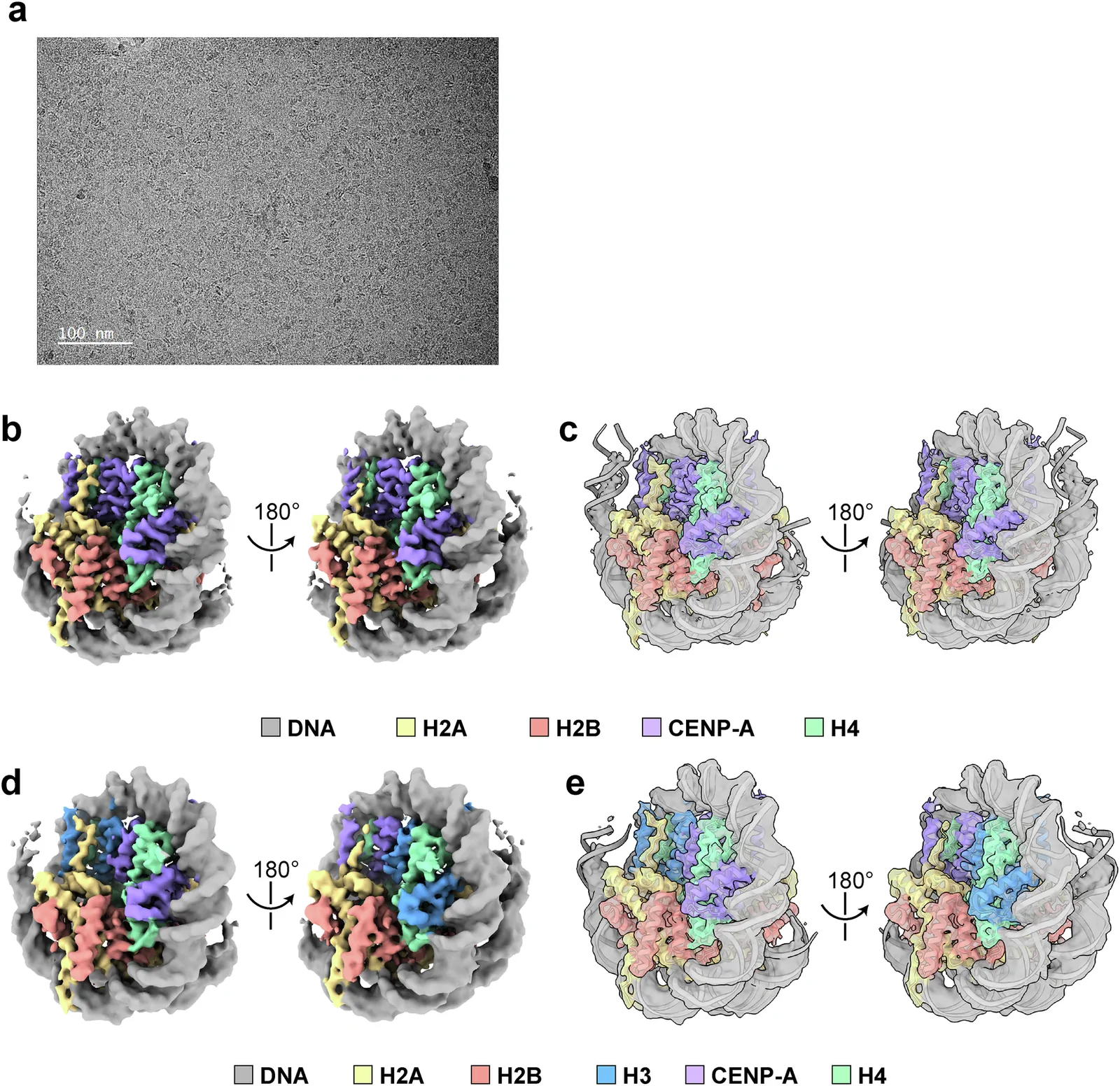
Cryo-EM structures of CENP-A nucleosomes isolated from human cells. **a** Representative cryo-EM micrograph of ChIP-derived CENP-A nucleosomes. **b** Structural overview of the CENP-A nucleosome at 3.12 Å resolution. **c** Overview of the alignment between the built model and the cryo-EM map at 3.12 Å resolution. **d** Structural overview of the CENP-A nucleosome at 3.58 Å resolution. **e** Overview of the alignment between the built model and the cryo-EM map at 3.58 Å resolution.

To visualize the architecture of human CENP-A nucleosomes, we subjected nucleosomes isolated from human cells to ChIP-CryoEM. Cryo-EM samples were prepared without crosslinking or nucleosome-stabilizing reagents, including single-chain variable fragments, to avoid artifacts that could obscure the native structural features. Cryo-EM micrographs revealed homogeneous particles corresponding to nucleosomes (Fig. 2a). Three-dimensional reconstruction yielded two maps of the cellular CENP-A nucleosomes at overall resolutions of 3.12 and 3.58 Å, allowing atomic model building and comparisons with reconstituted CENP-A nucleosome structures reported previously (Fig. 2b–e, Table 1).

**Table 1:** Cryo-EM data collection, refinement, and validation statistics

	#1 CENP-A homotypic nucleosome (EMD-67818) (PDB: 21MD)	#2 CENP-A/H3 heterotypic nucleosome (EMD-67819) (PDB: 21ME)
*Data collection and processing*		
Magnification	81,000	81,000
Voltage (kV)	300	300
Electron exposure (e^–^/Å^2^)	60.7	60.7
Defocus range (μm)	−1.25 to −2.25	−1.25 to −2.25
Pixel size (Å)	1.06	1.06
Symmetry imposed	C1	C1
Initial particle images (no.)	1,648,704	1,648,704
Final particle images (no.)	108,858	48,170
Map resolution (Å)	3.12	3.58
FSC threshold	0.143	0.143
Map resolution range (Å)	2.744–8.526	3.274–10.37
*Refinement*		
Initial model used (PDB code)	6E0P	6E0P, 7VZ4
Model resolution (Å)	3.4	4.1
FSC threshold	0.5	0.5
Model resolution range (Å)	N/D	N/D
Map sharpening *B* factor (Å^2^)	−86.9	−96.1
*Model composition*		
Non-hydrogen atoms	8860	8498
Protein residues	752	738
Ligands	262 (DN model)	242 (DN model)
*B* *factors* (*Å*^*2*^)		
Protein	98.75	147.91
Ligand	200.83 (DN model)	232.82 (DN model)
*R.m.s. deviations*		
Bond lengths (Å)	0.055	0.054
Bond angles (°)	2.173	2.110
*Validation*		
MolProbity score	1.49	1.51
Clashscore	4.46	6.03
Poor rotamers (%)	0.00	0.00
*Ramachandran plot*		
Favored (%)	96.06	96.95
Allowed (%)	3.94	3.05
Disallowed (%)	0.00	0.00

### Cryo-EM structure of the nucleosome containing two CENP-A molecules

**Fig. 3:**
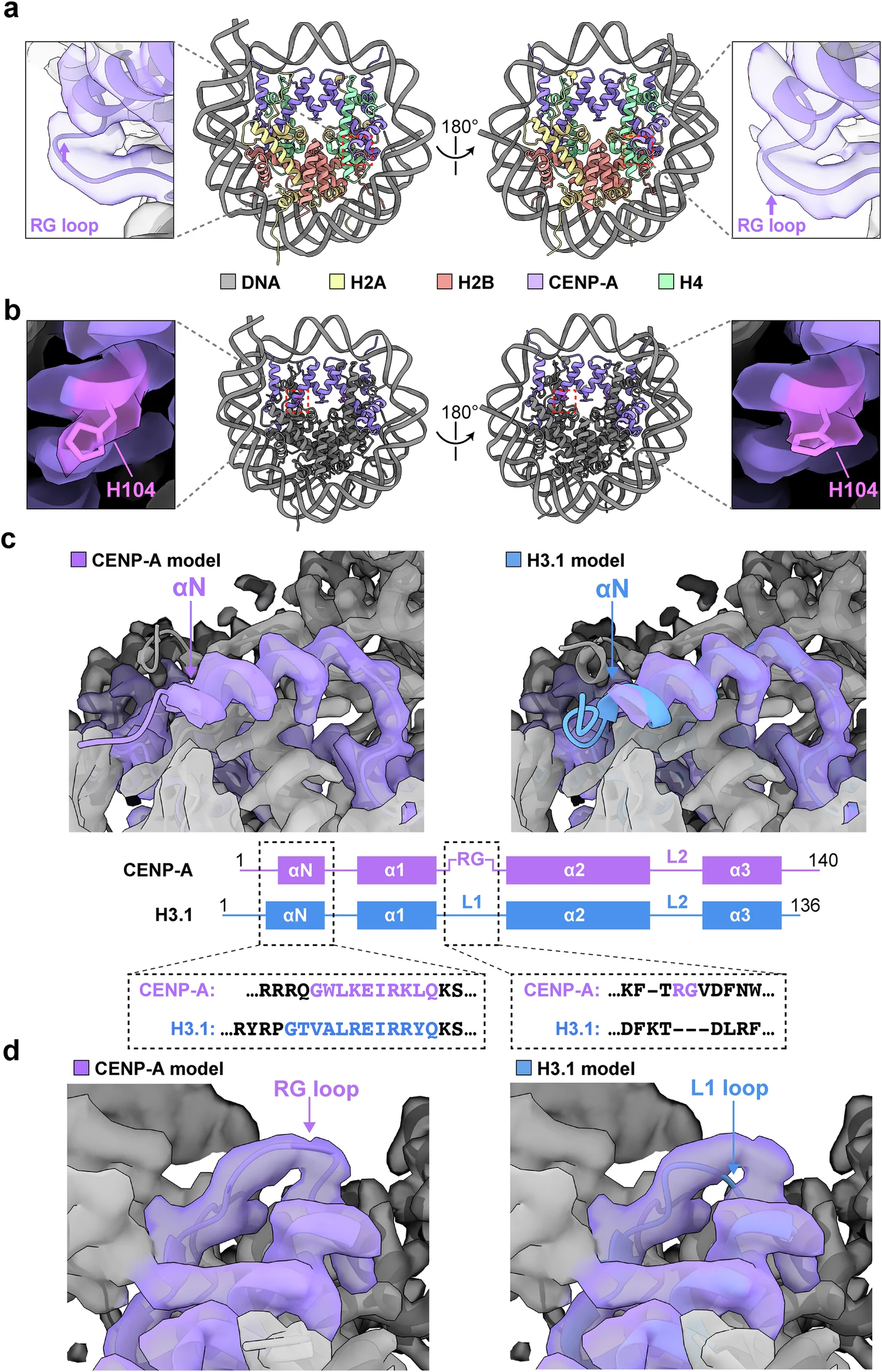
Cryo-EM structure of the homotypic CENP-A nucleosome. **a** Overview of the homotypic CENP-A nucleosome model and alignment of the RG loop and αN helix between the model and the cryo-EM map. **b** Alignment between the structural model and the density map around the H104 residue. **c** Comparison of the αN helix in the homotypic CENP-A nucleosome (density map and model) with that in the canonical nucleosome (PDB: 7VZ4). **d** Comparison of the RG loop in the homotypic CENP-A nucleosome (density map and model) with that in the canonical nucleosome (PDB: 7VZ4).

In the 3.12 Å structure, the density corresponding to the histone core clearly supported an octameric organization composed of two copies each of CENP-A, H2A, H2B, and H4 (Fig. 3a). The local density around CENP-A His104 supports the assignment of the reconstructed nucleosome as a CENP-A-containing nucleosome, because the corresponding residue is Gly in both canonical H3.1 and H3.3. Thus, the observed side-chain density at this position is consistent with the CENP-A-specific His104 residue and argues against the assignment as a canonical H3.1- or H3.3-containing nucleosome (Fig. 3b). Given that CENP-A is reportedly incorporated homotypically into centromeric chromatin^22,23^, it is possible that the homotypic CENP-A nucleosome observed here represents a CENP-A nucleosome assembled on centromeric α-satellite DNA.

Intriguingly, the cryo-EM structure revealed hallmark features that distinguish CENP-A nucleosomes from their canonical counterparts. The CENP-A N-terminal α-helix (Nα-helix) was markedly shorter than that of histone H3 (Fig. 3c). In addition, the CENP-A-specific RG loop within the histone fold extended beyond the outer surface of the DNA gyre, thereby positioned for access by centromere-specific binding factors, such as CENP-N (Fig. 3d)^35–38^. These features closely resemble those observed in reconstituted CENP-A nucleosomes, indicating that the defining structural characteristics are preserved in the cellular context. The exposed RG loop likely provides a structural platform for CCAN assembly, supporting its role in kinetochore formation.

### Cryo-EM structure of the heterotypic nucleosome containing one CENP-A histone and one H3 histone

**Fig. 4:**
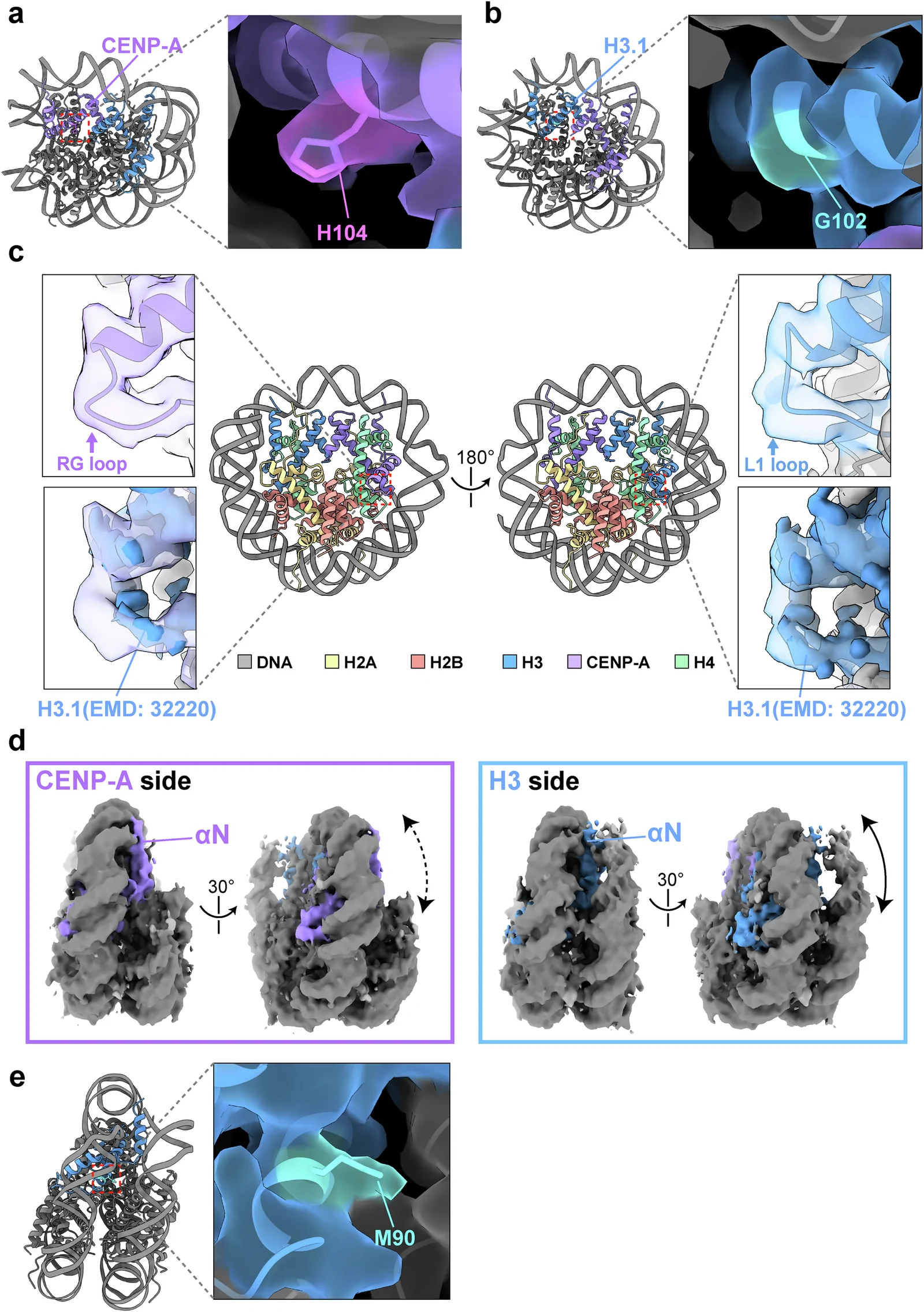
Cryo-EM structure of the heterotypic CENP-A/H3 nucleosome. **a** Alignment between the heterotypic CENP-A/H3 nucleosome model and the density map around the CENP-A H104 residue. **b** Alignment between the heterotypic CENP-A/H3 nucleosome model and the density map around the H3 G102 residue. **c** Overview of the heterotypic CENP-A/H3 nucleosome model and alignment of the RG loops between the model and the cryo-EM map. The density corresponding to the histone H3 L1 region on one side of the nucleosome disk is well fitted by the CENP-A model, including the RG loop, whereas the density on the opposite side is well fitted by the H3 model. This is supported by a comparison of the EM densities for the RG and L1 loops in the 3.58 Å structure with those of the reconstituted canonical nucleosome (EMD-32220). **d** Comparison of DNA lengths on each side of the nucleosome. The DNA segment indicated by the solid arrow is longer than that indicated by the dashed arrow. The DNA contacting the Nα-helix of CENP-A is shorter than that contacting the Nα-helix of H3. **e** Alignment between the heterotypic CENP-A/H3 nucleosome model and the density map around the H3.1 M90 residue.

The 3.58 Å reconstruction showed local asymmetry in the histone core and included density features more consistent with H3 than CENP-A on one side of the nucleosome. Although the local density does not allow unequivocal residue-level assignment in all regions, these map features, together with comparative model fitting, support the interpretation that this reconstruction is consistent with an asymmetric CENP-A/H3 heterotypic nucleosome. Notably, the CENP-A-specific His104 residue was only observed on one side (Fig. 4a). The corresponding residue of H3 is the Gly102 residue, which lacks a side chain moiety (Fig. 4b). Therefore, the peptide containing the His104 residue could be CENP-A. Consistently, the CENP-A-specific RG loop map was only seen on the CENP-A side, and the corresponding loop map on other side fit well with the H3 L1 loop (Fig. 4c). In addition, the DNA ends on the CENP-A side were not resolved, whereas those on the H3 side were visible (Fig. 4d). These observations are consistent with the interpretation that the 3.58 Å structure represents a heterotypic CENP-A/H3 nucleosome^24^. Given that heterotypic CENP-A/H3 nucleosomes reportedly form at ectopic loci apart from centromeres when CENP-A production is enhanced in aggressive cancer cells^23^, the heterotypic CENP-A/H3 nucleosome structure obtained from cellular human chromatin could be consistent with an ectopic CENP-A nucleosome on chromosome arms.

In the H3 molecule of the heterotypic CENP-A/H3 nucleosome, we observed side chain density corresponding to the H3.1-specific Met90 residue, supporting the presence of H3.1 in this nucleosome (Fig. 4e). However, this observation does not exclude the coexistence of heterotypic nucleosomes containing H3.3, as previously reported^23^. Because the corresponding residue at position 90 in H3.3 is glycine, which lacks a side chain moiety, the incorporation of H3.3 may not be detected based on side chain density in the present cryo-EM map, if it coexists with H3.1. Focused 3D classification using a soft mask restricted to the histone core, excluding the DNA region, may improve the local resolution around residue 90 and help distinguish H3.1- and H3.3-containing heterotypic nucleosome subclasses in future analyses.

Together, these findings establish that the human CENP-A nucleosome adopts a stable octameric architecture in vivo with both homotypic and heterotypic compositions and retains all hallmark structural characteristics previously attributed to CENP-A nucleosomes in vitro. These in-cell structures provide important insights into resolving long-standing controversies regarding the composition and conformation of centromeric nucleosomes in vivo and establish a structural framework for the maintenance of centromere identity.

## Discussion

The centromere has long served as a paradigm of epigenetic inheritance, with CENP-A nucleosomes representing the molecular mark that specifies its identity. Despite decades of investigation, how CENP-A nucleosomes are structurally organized within native chromatin remains incompletely understood. A major advance of this study is the application of ChIP-CryoEM to directly isolate and visualize CENP-A-containing nucleosomes from human cells. By combining chromatin immunoprecipitation with cryo-EM, this approach provides a proof-of-concept for determining the structures of selected chromatin particles in their cellular context, rather than in reconstituted systems^39,40^. This technical advance is particularly relevant to the long-standing question of whether cellular CENP-A nucleosomes predominantly adopt a canonical octameric organization or alternative non-canonical architectures.

Using the ChIP-CryoEM approach, we obtained the structures of CENP-A nucleosomes formed in human cells. The atomic model reveals a stable homotypic, octameric organization containing two copies each of H2A, H2B, CENP-A, and H4, while displaying the hallmark features previously inferred from in vitro studies, such as the shortened N-terminal α-helix and the distinctive RG loop^27–32^. We also obtained the structure of the heterotypic CENP-A/H3 nucleosome, which reportedly forms ectopic chromosome loci in aggressive cancer cells^23,24^. These findings are consistent with the idea that the defining structural characteristics of CENP-A nucleosomes are retained in vivo within a predominantly canonical octameric framework. At the same time, our results do not exclude the possibility that minor populations of non-canonical conformations may arise transiently or under specific cellular conditions. Indeed, such conformational heterogeneity has been suggested by previous atomic force microscopy and fluorescence correlation spectroscopy studies^17,18^, raising the possibility that the alternative states may be short-lived, cell cycle-regulated, or context-dependent. Future analyses with higher temporal resolution or under defined physiological conditions will be required to determine whether transient non-canonical states contribute to centromere dynamics.

Beyond the results reported here, the ability to capture cellular CENP-A nucleosome structures using ChIP-CryoEM opens a new avenue toward structural studies for other epigenetically defined chromatin states. Applying this approach across different cell cycle stages, developmental contexts, or disease states may reveal whether transient conformational variations of the CENP-A nucleosome contribute to centromere plasticity, stability, or dysfunction. In the future, ChIP-CryoEM may also enable direct investigations of the existence and functional significance of non-canonical CENP-A nucleosomes, including states that may exist only transiently or under specific physiological or pathological conditions. More broadly, ChIP-CryoEM provides a framework for determining the in vivo structures of selected chromatin-associated particles that have previously been accessible only in reconstituted systems, establishing a foundation for structural studies of genome organization and function in cells.

The CENP-A nucleosome structures obtained in this study also serve as a cellular structural reference for considering how CENP-A-specific nucleosome conformations potentially contribute to centromere identity. The partially unwrapped DNA termini and the CENP-A-specific structural motifs may confer flexibility and recognition properties important for the selective recruitment of centromeric factors, such as CENP-N, CENP-C, and other CCAN components^41–52^. DNA unwrapping of the CENP-A nucleosome was reportedly enhanced in the presence of CENP-B, although CENP-B itself was not visualized in the previous cryo-EM study^53^. The conformation of the CENP-A-specific RG-loop may provide the CENP-N binding sites^35–38^. The present cellular CENP-A nucleosome structures provide a starting framework for future studies of important issues, such as how CENP-A nucleosomes are precisely positioned at centromeres and how centromeric chromatin is organized into higher-order structures and maintained through cell divisions.

### Study limitations

It should be noted that this study has several limitations. First, CENP-A nucleosomes were purified from cells overexpressing FLAG-tagged CENP-A, which enabled the structural analysis but also likely produced both centromeric and ectopic CENP-A nucleosomes. We assigned the homotypic CENP-A nucleosome as a likely centromeric species and the heterotypic CENP-A/H3 nucleosome as a likely ectopic species based on their genome-wide distributions and previous studies, rather than directly identifying the genomic origin of each particle. Second, our ChIP-CryoEM analyses were performed with unsynchronized cells. In addition, the purification workflow involved MNase digestion, high-salt extraction, and immunoprecipitation. These procedures may select stable CENP-A-containing nucleosomes that are recoverable under these conditions. The development of milder, cell-cycle-specific sample preparation techniques using endogenous tagging will be crucial for demonstrating the existence of transient, low-abundance, cell-cycle-dependent, or context-specific non-canonical CENP-A particles in vivo.

## Methods

### Purification of CENP-A nucleosome from Expi293 cell nuclei

The Expi293F cells (Thermo Fisher Scientific) were grown in suspension culture at a density of approximately 3 × 10^6^ cells/ml using HE400AZ medium (GMEP), and Flag-tagged CENP-A was overexpressed with a pCMV-based expression plasmid using PEI MAX (Polysciences) and Opti-MEM reduced-serum medium (Thermo Fisher Scientific). Three days after transfection, the cells were collected by centrifugation at 400×*g* for 5 min at 4 °C, washed three times with PBS, and stored at −80 °C.

Collected cells were resuspended in CSK buffer (10 mM PIPES, 0.1% Triton X-100, 3 mM MgCl₂, 1 mM EGTA, 150 mM NaCl, and 1× Protease Inhibitor cocktail (Nacalai Tesque)) and incubated on ice for 30 min. After incubation, the suspension was centrifuged at 16,000×*g* for 10 min at 4 °C, and the supernatant was discarded. The nuclear pellet was washed twice with buffer 1 (50 mM HEPES–NaOH (pH 7.5), 100 mM NaCl, 0.3% NP-40) and then resuspended in buffer 1 supplemented with 1 mM CaCl₂. The suspension was vortexed and sonicated, and after the addition of MNase, it was incubated at 37 °C for 30 min. The reaction was quenched by adding EDTA to a final concentration of 1 mM. The mixture was then centrifuged at 16,000×*g* for 10 min at 4 °C, and the supernatant was discarded. The pellet was washed twice with buffer 1 (50 mM HEPES-NaOH (pH 7.5), 100 mM NaCl, 0.3% NP-40) and then resuspended in buffer 2 (50 mM HEPES–NaOH (pH 7.5), 400 mM NaCl, 0.3% NP-40) supplemented with 1 mM CaCl₂. The suspension was vortexed and sonicated, and after the addition of MNase, it was incubated at 37 °C for 30 min. The reaction was quenched by adding EDTA to a final concentration of 1 mM. The mixture was then centrifuged at 16,000×*g* for 10 min at 4 °C, and the supernatant was collected as the solubilized chromatin fraction. Chromatin digestion was confirmed by agarose gel electrophoresis with ethidium bromide staining (Supplementary Fig. 1a).

The solubilized chromatin fraction was mixed with Anti-FLAG affinity gel (Sigma), which had been pre-washed twice with buffer 3 (50 mM HEPES–NaOH (pH 7.5), 400 mM NaCl), and incubated with rotation at 4 °C for 2 h. The suspension was then centrifuged at 4000×*g* for 10 min at 4 °C, and the supernatant was discarded as the unbound fraction. The remaining gel was washed twice with buffer 3, mixed with elution buffer (50 mM HEPES–NaOH (pH 7.5), 400 mM NaCl, and 100 μg/ml FLAG peptide), and incubated with rotation at 4 °C for 1 h. The suspension was centrifuged at 4000×*g* for 10 min at 4 °C, and the supernatant was collected as the eluted fraction. This elution step was performed once more. The two collected eluted fractions were combined and concentrated using an Amicon Ultra 30K filter (Millipore). The components of each fraction were confirmed by SDS–PAGE (Fig. 1b) and western blotting using an anti-CENP-A antibody (MBL, D115-3) and an anti-FLAG M2 antibody (Sigma, F3165) (Supplementary Fig. 1b, c).

### Cryo-EM sample preparation

The sucrose gradient was prepared using low buffer (10 mM HEPES–NaOH (pH 7.5), 1 mM DTT, 400 mM NaCl, and 10% sucrose) and high buffer (10 mM HEPES–NaOH (pH 7.5), 1 mM DTT, 400 mM NaCl, and 50% sucrose) by using a Gradient Master (SKB). The concentrated elution fraction was applied on top of the sucrose gradient and centrifuged at 35,000 rpm for 21 h at 4 °C. After centrifugation, 700 µl fractions were collected from the top of the gradient. A 10 µl portion of each fraction was treated with proteinase K to digest histones, and the DNA lengths were examined by 1.5% agarose gel electrophoresis with ethidium bromide staining (Fig. 1c). The protein components of fractions 5–9, which appeared to contain mono-, di-, and tri-nucleosomes based on their DNA lengths, were analyzed by SDS–PAGE (Fig. 1d) and western blotting using an anti-CENP-A antibody (MBL, D115-3) and an anti-FLAG M2 antibody (Sigma, F3165) (Supplementary Fig. 1d, e). Fractions 5 and 6 were subjected to buffer exchange into buffer 3 using a PD MiniTrap G-25 column (Cytiva), and the CENP-A nucleosome sample was concentrated to a final volume of 50 µl using an Amicon Ultra 30 K filter (Millipore).

### Sequencing sample preparation

Fractions 5 and 6 were further treated with proteinase K to digest proteins, and the remaining DNA was purified using a DNA Clean & Concentrator-5 kit (Zymo Research).

### Next-generation sequencing analyses

The paired-end ChIP-seq library was prepared from ChIPed DNA, using a ThruPLEX® DNA-Seq Kit (TaKaRa, R400674). Sequencing was performed on an Illumina NovaSeq 6000 sequencer (Replicate 1) and an Illumina MiniSeq sequencer (Replicate 2). Adapter DNA sequences were trimmed using Trim Galore (version 0.6.10). The reads were mapped to the human genome (GRCh38) using Bowtie2 (version 2.5.4)^54^ with the option ‘-k 3‘. BAM files were created, and the PCR duplicates were removed using SAMtools (version 1.22.1)^55^. IGV tracks were created with bamCoverage from deepTools (version 3.5.6)^56^ using the reads with presumable lengths of nucleosomal DNA and the following options: ‘--binSize 100 --smoothLength 1000 --normalizeUsing CPM --centerReads --minFragmentLength 100 --maxFragmentLength 250‘.

### Cryo-EM grid preparation

Quantifoil Au R1.2/1.3 200-mesh grids, coated with a monolayer of graphene, were treated with a UV–ozone cleaner for 10 min to increase their hydrophilicity, and the concentrated sample was applied to the grids. The grids were plunge frozen in liquid ethane using a Vitrobot Mark IV (Thermo Fisher Scientific) at 4 °C and 100% humidity.

### Cryo-EM data collection and processing

Cryo-EM data were collected on a Krios G4 transmission electron microscope (Thermo Fisher Scientific) operated at 300 kV and equipped with a BioQuantum K3 camera (Gatan). A total of 5640 movies were recorded using EPU (Thermo Fisher Scientific) at a nominal magnification of ×81,000, corresponding to a pixel size of 1.06 Å, with defocus values ranging from –1.25 to –2.25 µm. Each movie was fractionated into 40 frames with a total electron dose of ~60 e^−^/Å². All subsequent data processing was performed using cryoSPARC v4.0.1^57–59^.

The detailed process is indicated in Supplementary Fig. 2. Patch motion correction was applied to the movie stacks, followed by contrast transfer function (CTF) estimation using Patch CTF. After curation, 5372 micrographs were retained. A total of 1,863,200 particles were automatically picked using blob picking with an applied particle diameter of 100–300 Å, and extracted with a box size of 256 pixels. After two rounds of 2D classification, ab-initio model reconstruction was performed, and the best resolved volume was used for the initial volume of heterogeneous refinement. Two well-resolved volumes were subjected to 3D classification to separate homotypic and heterotypic CENP-A/H3 nucleosomes, and the particles were divided into two groups based on the number of CENP-A molecules per nucleosome. Each group was further refined by additional rounds of 3D classification to select the final particle sets. Ultimately, the map of the homotypic CENP-A nucleosome was reconstructed at a resolution of 3.1 Å from 108,873 particles, and the map of the heterotypic CENP-A nucleosome was reconstructed at a resolution of 3.5 Å from 48,170 particles (Supplementary Fig. 3).

### Model building

For model building of the homotypic CENP-A nucleosome, the atomic model was generated using the cryo-EM structure of the reconstituted homotypic CENP-A nucleosome (PDB ID: 6E0P)^31^. For the heterotypic CENP-A/H3 nucleosome, the model was built using PDB 7VZ4^60^ together with the CENP-A model from the cryo-EM structure of the CENP-A nucleosome (PDB ID: 6E0P). The atomic coordinates were refined by phenix.real space refinement, followed by geometry minimization, and major clashes were manually corrected using Coot^61–64^.

### The use of large language models

ChatGPT, a large language model developed by OpenAI, was employed solely as a supportive tool during the manuscript preparation processes. Full responsibility for the content and conclusions of this work rests with the authors.

### Statistics and reproducibility

No statistical methods were used to predetermine sample sizes. Cryo-EM micrographs of insufficient quality were excluded based on ice quality, image drift, astigmatism, and estimated CTF resolution. The sequencing experiments were independently repeated twice and yielded similar results. No randomization or blinding was applied.

## Supplementary information

Transparent Peer Review file

Supplementary Information

## Data Availability

The electron density maps and corresponding atomic coordinates have been deposited in the Electron Microscopy Data Bank (EMDB) and the Protein Data Bank (PDB). The CENP-A homotypic nucleosome structure has been deposited under PDB accession code 21MD and EMDB accession code EMD-67818, and the CENP-A/H3 heterotypic nucleosome structure under PDB accession code 21ME and EMDB accession code EMD-67819. ChIP-seq data generated in this study have been deposited in the Gene Expression Omnibus under accession No. GSE315822.
