# Supplementary material for: Cryo-EM structures of CENP-A nucleosomes isolated from human cells
Source: Commun Biol. 2026 Sep 29;9:1253. doi: 10.1038/s42003-026-10946-x (PMC13623831; doi:10.1038/s42003-026-10946-x)
Supplement: Supplementary file 1 [file 42003_2026_10946_MOESM1_ESM.pdf]

nature portfolio

# Peer Review File

# Cryo-EM structures of CENP-A nucleosomes isolated from human cells

Corresponding Author: Professor Hitoshi Kurumizaka

This manuscript has been previously reviewed at another journal. This document only contains information relating to versions considered at Communications Biology.

**This file contains all reviewer reports in order by version, followed by all author rebuttals in order by version.**

Version 0:

Reviewer comments:

Reviewer #1

(Remarks to the Author)
I appreciate the authors' careful revision of the manuscript. As stated in my previous review, this study uses ChIP-CryoEM on cell-derived human CENP-A nucleosomes to address a long-standing question in centromere biology. The topic is broadly important and relevant, since CENP-A chromatin is vital to centromere identity and chromosome segregation. An important strength of the study is the ability to obtain relatively high-resolution structures from purified cell-derived particles containing CENP-A. The work provides convincing evidence that octameric CENP-A nucleosomes can be extracted from human cells, possibly in both homotypic and heterotypic forms.

In my view, the revised version is more appropriate for a specialized journal such as Communications Biology. The authors have softened and better framed the scope of the conclusions allowed by the data, and they now acknowledge more clearly the limitations and implications of the study, including the use of overexpressed FLAG-tagged CENP-A, the mixed nature of the purified population, and the more cautious interpretation of the heterotypic class. I also appreciate the clarifications made regarding the map interpretation and structural assignments, which address my previous concerns in those areas. These are meaningful improvements over the previous version.
Overall, I think the manuscript has improved significantly and is now better aligned with the scope of the data. I therefore support publication in its current form.

Reviewer #2

(Remarks to the Author)
I co-reviewed this manuscript with one of the reviewers who provided the listed reports. This is part of the Communications Biology initiative to facilitate training in peer review and to provide appropriate recognition for Early Career Researchers who co-review manuscripts.

Reviewer #3

(Remarks to the Author)
In this revision, the authors have responded to referees' comments by widely softening their language and claims throughout the manuscript to more accurately represent the findings and significance of this paper. The authors have satisfactorily responded to my comments and made improvements to their figures. With these edits, I feel that this manuscript is appropriate for publication in Communications Biology.

While this manuscript does not represent a large advance (and the authors do not add new experiments or major new analysis in their revision), the work is scientifically sound and it does make worthwhile contributions including a proof-of-concept of using ChIP-CryoEM for CENP-A to structurally characterize ex vivo CENP-A nucleosomes as well as a data point added to the evidence in favor of octameric CENP-A nucleosomes. Most importantly, the text of the manuscript now appropriately represents and characterizes these advances.

Minor comments:
1. Lines 31-33 in the abstract ("how this epigenetic mark is structurally encoded within native chromatin has remained a long-standing and unresolved controversy") still feels a little over-stated and should be edited. I believe the authors are trying to refer to the octamer vs. tetramer controversy here, but this is not clear. And, in general, the structure of CENP-A nucleosomes within chromatin is more of a gap in scientific understanding than a controversy. It would be better to say that the structure of CENP-A nucleosomes in native chromatin "remains incompletely understood" or something along those lines.
2. The discussion would be more impactful if framed more prominently around the technical advance/proof-of-concept of ChIP-CryoEM of CENP-A. In its current form the discussion only mentions this starting at line 228. The preceding two paragraphs, starting at lines 207 and 220, are weak. Specifically, the authors claim that this work provides "a molecular basis" for understanding higher order chromatin structure and that their findings "refine current models of centromere propagation", both of which are a bit of a stretch. However, the potential for future work building on this technique (discussed in the paragraph starting at line 228) is very real and has the potential for large impact. I would suggest emphasizing this point and perhaps framing the other discussion topics around this. If the authors do not like this suggestion, then regardless I would recommend softening the wording of paragraphs 207 and 220.
3. There are several minor typographic (missing spaces, etc) and grammatical errors throughout the text that could be addressed at copyediting.

Reviewer #4

(Remarks to the Author)
Methodological Limitations
The authors have appropriately revised the text to acknowledge the limitations of their technique, specifically regarding the use of overexpressed CENP-A and an extraction process that may selectively favor stable complexes while overlooking transient or less stable in vivo assemblies.
Correction of Selective Citation
While the manuscript has been revised, the citation of prior structural work remains highly selective. The authors build upon an extensive foundation of in vitro data but credit very little of it, with preference to their own work. This "cherry-picking" of references obscures the decade of foundational characterization provided by multiple independent laboratories.
To ensure academic rigor and distinguish new findings from established knowledge, the authors must explicitly credit the following milestones:
CENP-A/H4 tetramers, nucleosomes, and sub-nucleosomes: (PMID 20739937, 27499292, 30718488, 32313946).
Non-nucleosomal CENP-A assemblies: (PMID 41397873).
Complexes with CENP-C: (PMID 25954010, 26878239, 31475439, 33463726).
Complexes with CENP-N: (PMID 29350209, 35422519).
Complexes with CENP-B: (PMID: 38086807)
Full CCAN-CENP-A assemblies (Human and Yeast): (PMID 35420891, 35525244; PMID 31578520).
These studies were instrumental in demonstrating how the unique features of the CENP-A nucleosome—including its histone composition, DNA-end flexibility, and specialized surface properties (such as the L1 loop and C-terminal tail)—provide the physical platform for CCAN binding. It is this specific recruitment and assembly of the CCAN that licenses centromere function and ensures high-fidelity chromosome segregation.
While these works collectively support the stability of the octameric CENP-A nucleosome, they do not preclude the existence of non-nucleosomal structures in a cellular context.

Reviewer #5

(Remarks to the Author)
The authors have made substantial textual revisions that address most of the concerns raised in the previous review, particularly by tempering their interpretations and conclusions to better align with the presented data. Overall, I believe the revised manuscript is, in principle, suitable for publication in Communications Biology.
However, given the limitations associated with the techniques and experimental design, I recommend that the authors more explicitly acknowledge these limitations, particularly with respect to the extent to which their data can address the ongoing controversy surrounding the nature of native CENP-A nucleosomes in vivo. In addition to the revisions incorporated throughout the Results section, it may be more appropriate to include a dedicated paragraph in the Discussion or add a separate "Study Limitations" section at the end of the main text. Doing so would help readers place the findings in the broader context of current centromere biology and the existing body of evidence in the field.

Version 1:

Reviewer comments:

Reviewer #4

(Remarks to the Author)
Authors have addressed all my comments.

Reviewer #5

(Remarks to the Author)
The authors have satisfactorily addressed all reviewer concerns and the manuscript is now suitable for publication at Communications Biology.

**Open Access** This Peer Review File is licensed under a Creative Commons Attribution 4.0 International License, which permits use, sharing, adaptation, distribution and reproduction in any medium or format, as long as you give appropriate credit to the original author(s) and the source, provide a link to the Creative Commons license, and indicate if changes were made.
In cases where reviewers are anonymous, credit should be given to 'Anonymous Referee' and the source.
The images or other third party material in this Peer Review File are included in the article's Creative Commons license, unless indicated otherwise in a credit line to the material. If material is not included in the article's Creative Commons license and your intended use is not permitted by statutory regulation or exceeds the permitted use, you will need to obtain permission directly from the copyright holder.
To view a copy of this license, visit https://creativecommons.org/licenses/by/4.0/

reviewers' comments:

Reviewer #1 (Remarks to the Author):

I appreciate the authors' careful revision of the manuscript. As stated in my previous review, this study uses ChIP-CryoEM on cell-derived human CENP-A nucleosomes to address a long-standing question in centromere biology. The topic is broadly important and relevant, since CENP-A chromatin is vital to centromere identity and chromosome segregation. An important strength of the study is the ability to obtain relatively high-resolution structures from purified cell-derived particles containing CENP-A. The work provides convincing evidence that octameric CENP-A nucleosomes can be extracted from human cells, possibly in both homotypic and heterotypic forms.

In my view, the revised version is more appropriate for a specialized journal such as Communications Biology. The authors have softened and better framed the scope of the conclusions allowed by the data, and they now acknowledge more clearly the limitations and implications of the study, including the use of overexpressed FLAG-tagged CENP-A, the mixed nature of the purified population, and the more cautious interpretation of the heterotypic class. I also appreciate the clarifications made regarding the map interpretation and structural assignments, which address my previous concerns in those areas. These are meaningful improvements over the previous version.
Overall, I think the manuscript has improved significantly and is now better aligned with the scope of the data. I therefore support publication in its current form.

Reply)
Thank you very much for your favorable comments.
We appreciate your evaluations.

Reviewer #2 (Remarks to the Author):

I co-reviewed this manuscript with one of the reviewers who provided the listed reports. This is part of the Communications Biology initiative to facilitate training in peer review and to provide appropriate recognition for Early Career Researchers who co-review manuscripts.

Reviewer #3 (Remarks to the Author):

In this revision, the authors have responded to referees' comments by widely softening their language and claims throughout the manuscript to more accurately represent the findings and significance of this paper. The authors have satisfactorily responded to my comments and made improvements to their figures. With these edits, I feel that this manuscript is appropriate for publication in Communications Biology.

While this manuscript does not represent a large advance (and the authors do not add new experiments or major new analysis in their revision), the work is scientifically sound and it does make worthwhile contributions including a proof-of-concept of using ChIP-CryoEM for CENP-A to structurally characterize ex vivo CENP-A nucleosomes as well as a data point added to the evidence in favor of octameric CENP-A nucleosomes. Most importantly, the text of the manuscript now appropriately represents and characterizes these advances.

Minor comments:

1. Lines 31-33 in the abstract ("how this epigenetic mark is structurally encoded within native chromatin has remained a long-standing and unresolved controversy") still feels a little over-stated and should be edited. I believe the authors are trying to refer to the octamer vs. tetramer controversy here, but this is not clear. And, in general, the structure of CENP-A nucleosomes within chromatin is more of a gap in scientific understanding than a controversy. It would be better to say that the structure of CENP-A nucleosomes in native chromatin "remains incompletely understood" or something along those lines.

Reply)

We thank the reviewer for this helpful suggestion. Accordingly, we have revised the sentence in the Abstract to avoid framing this issue as a controversy and to more accurately reflect the current state of knowledge. The revised text now reads:
"Although the structures of reconstituted CENP-A nucleosomes have provided important insights, how this epigenetic mark is structurally organized within native chromatin remains incompletely understood."

2. The discussion would be more impactful if framed more prominently around the

technical advance/proof-of-concept of ChIP-CryoEM of CENP-A. In its current form the discussion only mentions this starting at line 228. The preceding two paragraphs, starting at lines 207 and 220, are weak. Specifically, the authors claim that this work provides “a molecular basis” for understanding higher order chromatin structure and that their findings “refine current models of centromere propagation”, both of which are a bit of a stretch. However, the potential for future work building on this technique (discussed in the paragraph starting at line 228) is very real and has the potential for large impact. I would suggest emphasizing this point and perhaps framing the other discussion topics around this. If the authors do not like this suggestion, then regardless I would recommend softening the wording of paragraphs 207 and 220.

Reply)
We thank the reviewer for this constructive suggestion. We agree that the Discussion should more prominently emphasize the technical advance and proof-of-concept aspect of applying ChIP-CryoEM to CENP-A nucleosomes. We have therefore revised the Discussion to introduce this point earlier and to frame the biological implications in the context of what this approach now makes possible.

3. There are several minor typographic (missing spaces, etc) and grammatical errors throughout the text that could be addressed at copyediting.

Reply)
The final version of the manuscript has been checked by an English editing service.

Reviewer #4 (Remarks to the Author):

Methodological Limitations
The authors have appropriately revised the text to acknowledge the limitations of their technique, specifically regarding the use of overexpressed CENP-A and an extraction process that may selectively favor stable complexes while overlooking transient or less stable in vivo assemblies.
Correction of Selective Citation
While the manuscript has been revised, the citation of prior structural work remains highly selective. The authors build upon an extensive foundation of in vitro data but credit very little of it, with preference to their own work. This "cherry-picking" of

references obscures the decade of foundational characterization provided by multiple independent laboratories.

To ensure academic rigor and distinguish new findings from established knowledge, the authors must explicitly credit the following milestones:

CENP-A/H4 tetramers, nucleosomes, and sub-nucleosomes: (PMID 20739937, 27499292, 30718488, 32313946).

Non-nucleosomal CENP-A assemblies: (PMID 41397873).

Complexes with CENP-C: (PMID 25954010, 26878239, 31475439, 33463726).

Complexes with CENP-N: (PMID 29350209, 35422519).

Complexes with CENP-B: (PMID: 38086807)

Full CCAN-CENP-A assemblies (Human and Yeast): (PMID 35420891, 35525244; PMID 31578520).

These studies were instrumental in demonstrating how the unique features of the CENP-A nucleosome—including its histone composition, DNA-end flexibility, and specialized surface properties (such as the L1 loop and C-terminal tail)—provide the physical platform for CCAN binding. It is this specific recruitment and assembly of the CCAN that licenses centromere function and ensures high-fidelity chromosome segregation.

While these works collectively support the stability of the octameric CENP-A nucleosome, they do not preclude the existence of non-nucleosomal structures in a cellular context.

Reply)

In response to this suggestion, we have revised the manuscript to cite all of the publications recommended by the reviewer. We also rewrote the Discussion section to more clearly acknowledge the extensive body of prior *in vitro* structural work and to distinguish these established findings from the new insights provided by the present study. In particular, we now emphasize that the cellular CENP-A nucleosome structures presented here provide a starting framework for future studies addressing important questions, such as how CENP-A nucleosomes are precisely positioned at centromeres and how centromeric chromatin is organized into higher-order structures and maintained through cell divisions.

In addition, we have clarified that, although previous studies collectively support the stability of the octameric CENP-A nucleosome, they do not exclude the possible existence of non-nucleosomal or non-canonical CENP-A particles *in vivo*. We now state that the development of milder, cell-cycle-specific sample preparation techniques using endogenous tagging will be crucial for demonstrating the existence of

transient, low-abundance, cell-cycle-dependent, or context-specific non-canonical CENP-A particles *in vivo*.

Reviewer #5 (Remarks to the Author):

The authors have made substantial textual revisions that address most of the concerns raised in the previous review, particularly by tempering their interpretations and conclusions to better align with the presented data. Overall, I believe the revised manuscript is, in principle, suitable for publication in Communications Biology. However, given the limitations associated with the techniques and experimental design, I recommend that the authors more explicitly acknowledge these limitations, particularly with respect to the extent to which their data can address the ongoing controversy surrounding the nature of native CENP-A nucleosomes in vivo. In addition to the revisions incorporated throughout the Results section, it may be more appropriate to include a dedicated paragraph in the Discussion or add a separate "Study Limitations" section at the end of the main text. Doing so would help readers place the findings in the broader context of current centromere biology and the existing body of evidence in the field.

Reply)
According to this reviewer's suggestion, we have added a short "Study limitations" section at the end of the Discussion. This section now clarifies that our samples were prepared from cells overexpressing FLAG-tagged CENP-A, likely contain both centromeric and ectopic CENP-A nucleosomes, and were obtained from unsynchronized cells using a purification and cryo-EM classification workflow that may preferentially recover stable nucleosomal particles. We also clarify that our data support octameric architectures for the major CENP-A-containing nucleosomes recovered under these conditions, but do not exclude transient, low-abundance, cell-cycle-dependent, or context-specific non-canonical CENP-A particles *in vivo*.
