# Supplementary material for: Cryo-EM structures of CENP-A nucleosomes isolated from human cells
Source: Commun Biol. 2026 Sep 29;9:1253. doi: 10.1038/s42003-026-10946-x (PMC13623831; doi:10.1038/s42003-026-10946-x)
Supplement: Supplementary file 2 [file 42003_2026_10946_MOESM2_ESM.pdf]

Supplementary Information

Cryo-EM structures of CENP-A nucleosomes isolated from human cells

Eisho Tanida[1,2], Suguru Hatazawa[1], Takumi Oishi[1,2], Yuki Kobayashi[1], Mitsuo Ogasawara[1], Marta A. Jaskiewicz[1], Akihito Harada[3], Takeru Fujii[4], Yasuyuki Ohkawa[4], Yoshimasa Takizawa[1,5], and Hitoshi Kurumizaka[1,2,6,7]*

[1] Laboratory of Chromatin Structure and Function, Institute for Quantitative Biosciences, The University of Tokyo, 1-1-1 Yayoi, Bunkyo-ku, Tokyo 113-0032, Japan.

[2] Department of Biological Sciences, Graduate School of Science, The University of Tokyo, 1-1-1 Yayoi, Bunkyo-ku, Tokyo 113-0032, Japan.

[3] Department of Multi-Omics, Graduate School of Medical Sciences, Kyushu University, 3-1-1 Maidashi, Higashi-ku, Fukuoka 812-0054, Japan.

[4] Division of Transcriptomics, Medical Institute of Bioregulation, Kyushu University, 3-1-1 Maidashi, Higashi-ku, Fukuoka 812-0054, Japan.

[5] Department of Computational Biology and Medical Sciences, Graduate School of Frontier Sciences, The University of Tokyo, 1-1-1 Yayoi, Bunkyo-ku, Tokyo 113-0032, Japan.

[6] RIKEN Center for Integrative Medical Sciences, 1-7-22 Suehiro-cho, Tsurumi-ku, Yokohama 230-0045, Japan.

[7]Division of Chromatin Structure and Function, Medical Institute of Bioregulation, Kyushu University, 3-1-1 Maidashi, Higashi-ku, Fukuoka 812-0054, Japan.

*To whom correspondence should be addressed; E-mail address: kurumizaka@iqb.u-tokyo.ac.jp

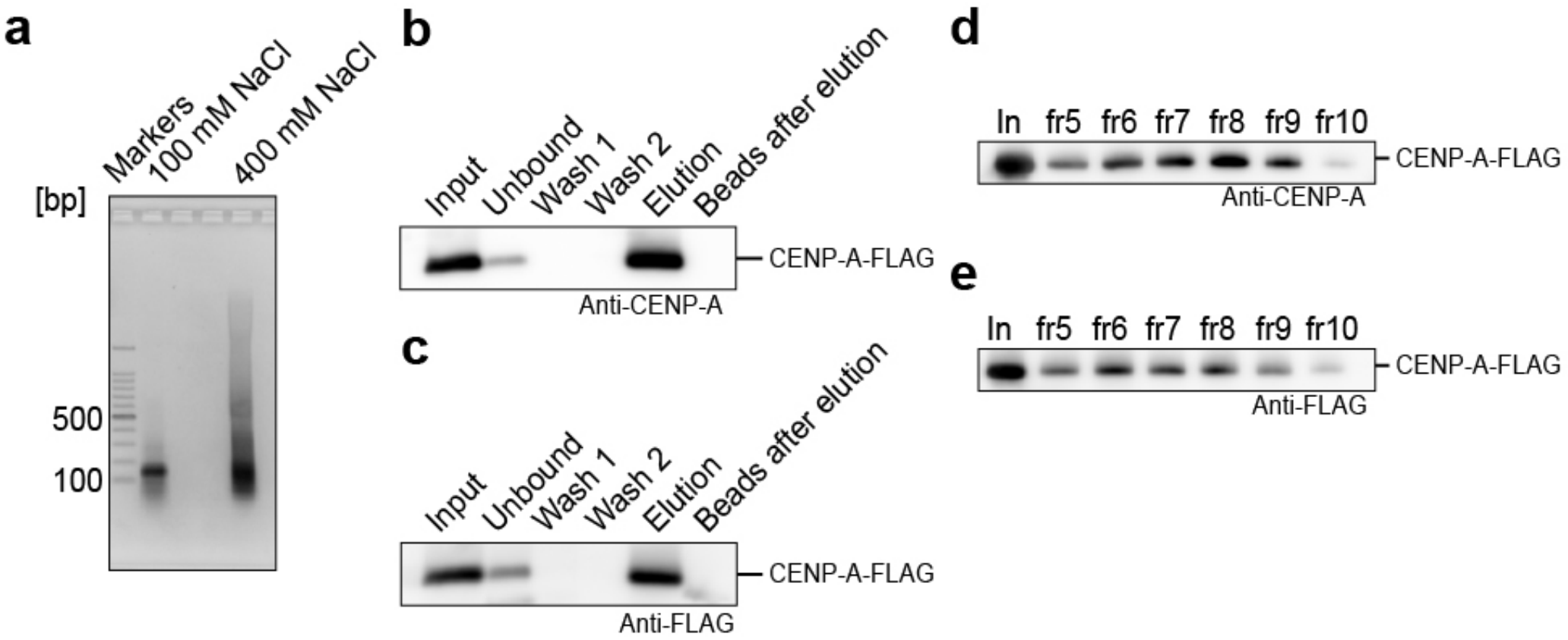

Supplementary Fig. 1 Purification of CENP-A nucleosome from EXPi293 cell nuclei

a, Chromatin digestion by micrococcal nuclease (MNase). Digested chromatin in first 100 mM and subsequent 400 mM NaCl conditions are shown. **b**, Western blot analysis of proteins in fractions from the FLAG immunoprecipitation (IP), using an anti–CENP-A antibody. **c**, Western blot analysis of proteins in fractions from the FLAG immunoprecipitation (IP), using an anti-FLAG antibody. **d**, Western blot analysis of proteins in fractions from the sucrose gradient experiment, using an anti–CENP-A antibody. **e**, Western blot analysis of proteins in fractions from the sucrose gradient experiment, using an anti-FLAG antibody.

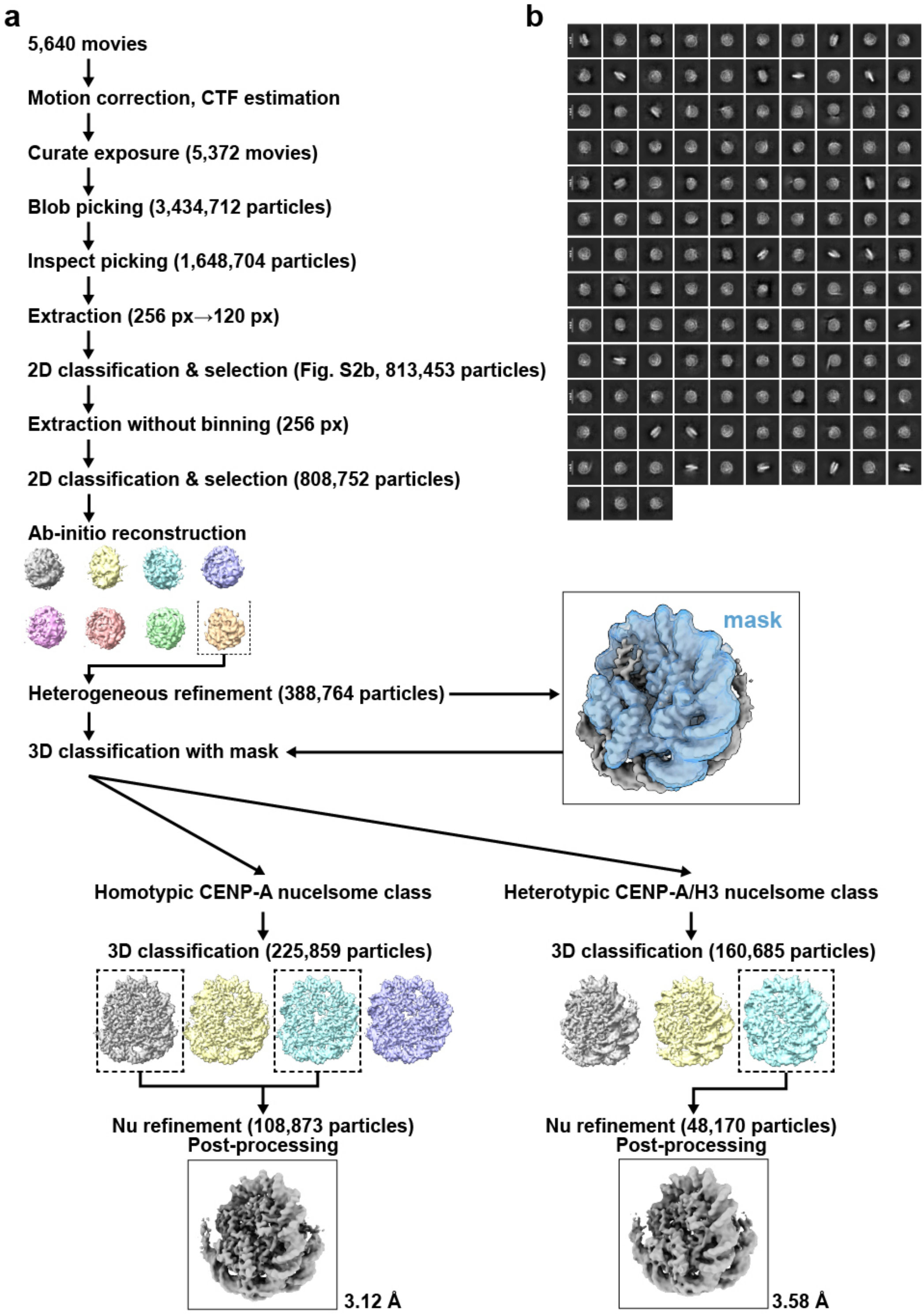

Supplementary Fig. 2 Cryo-EM data processing

a, Image processing workflow in cryoSPARC. **b**, Selected 2D class averages from 2D classification of extracted particles. The box size is 256 × 256 pixels.

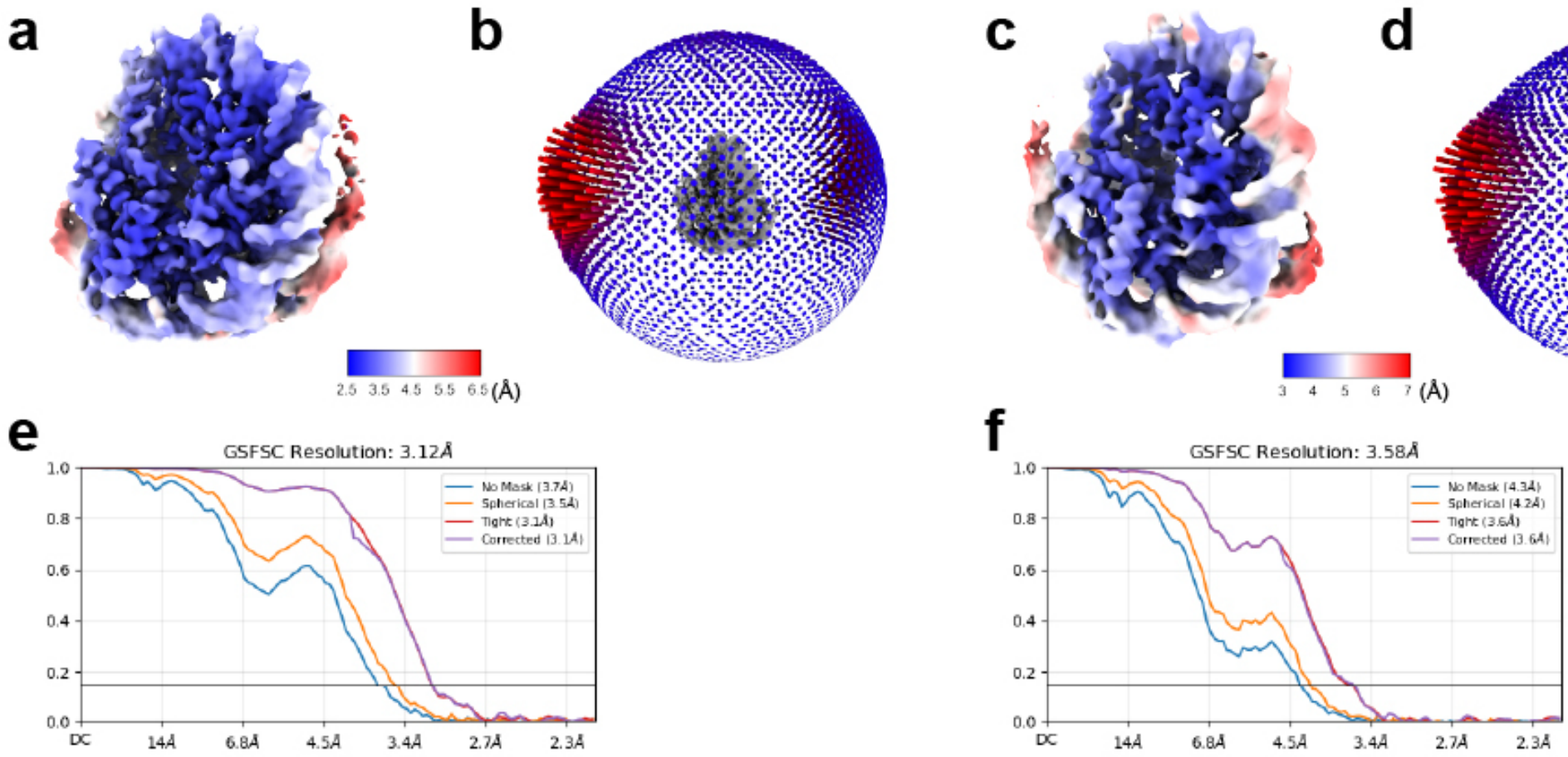

Supplementary Fig. 3 Quality of structures

a, Local resolution map of the homotypic CENP-A nucleosome at 3.12 Å resolution. **b**, Euler angle distribution of the 3.12 Å reconstruction. **c**, Local resolution map of the homotypic CENP-A nucleosome at 3.58 Å resolution. **d**, Euler angle distribution of the 3.58 Å reconstruction. **e**, Fourier shell correlation (FSC) curves for the 3.12 Å reconstruction. The resolution was estimated using the FSC = 0.143 criterion. **f**, Fourier shell correlation (FSC) curves for the 3.58 Å reconstruction. The resolution was estimated using the FSC = 0.143 criterion.

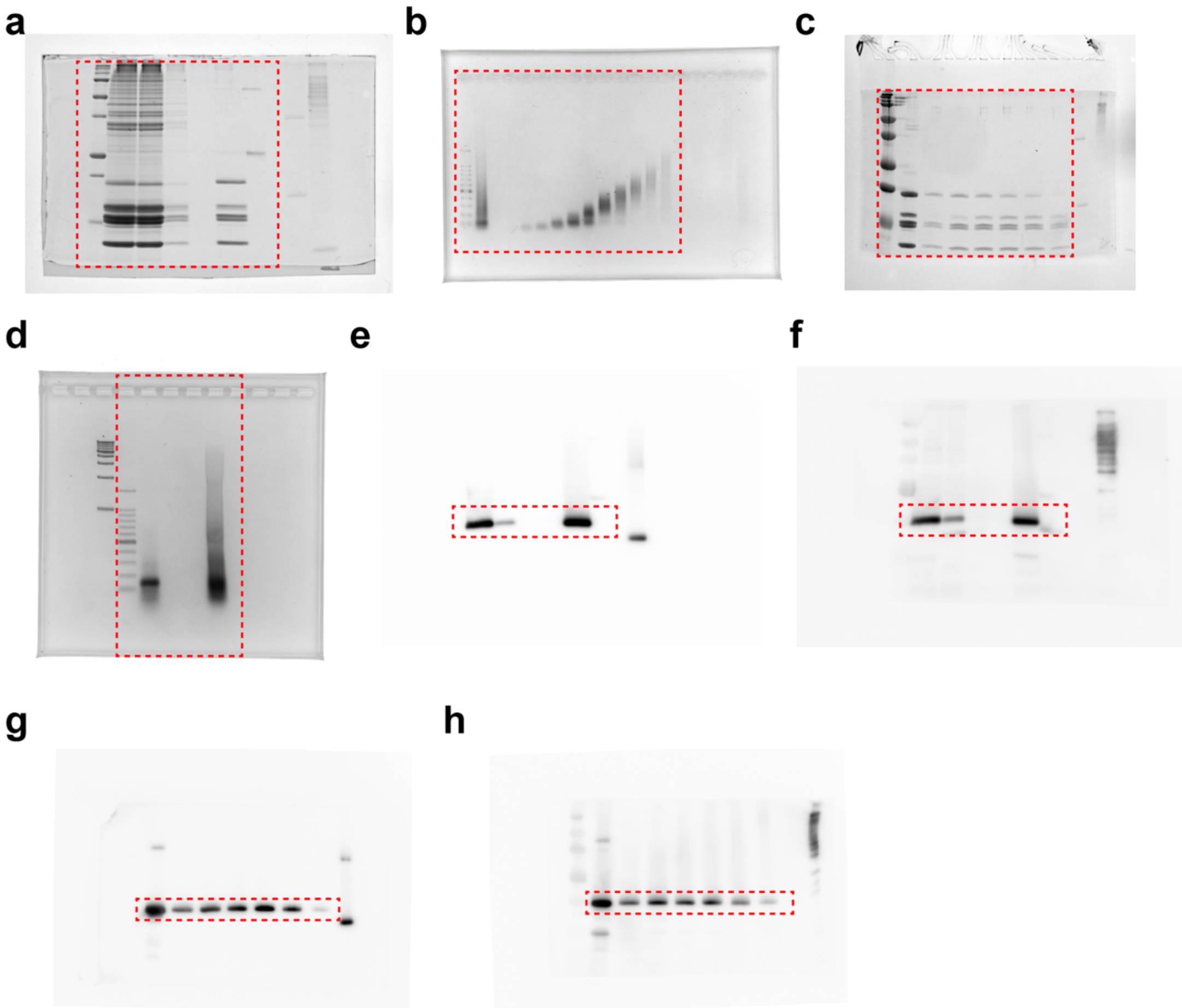

Supplementary Fig. 4 Uncropped images

a, Uncropped image of Fig. 1b. **b**, Uncropped image of Fig. 1c. **c**, Uncropped image of Fig. 1d. **d**, Uncropped image of Supplementary Fig. 1a. **e**, Uncropped image of Supplementary Fig. 1b. **f**, Uncropped image of Supplementary Fig. 1c. **g**, Uncropped image of Supplementary Fig. 1d. **h**, Uncropped image of Supplementary Fig. 1e.
